# Bone Lesions of Leprosy

**Published:** 1912-09

**Authors:** 


Bone Lesions of Leprosy.—A. B. Herrick and T. W. Earhardt, (*ibid.*) classify the bone changes as 1. Tropic, with simple atrophy and absorption; 2. Osteomyelitis or periostitis due to the bacillus; 3. Necrosis or inflammation of bone or periosteum due to secondary pyogens. 19 beautiful skiagrams illustrate the article, showing the various phases and stages.

Murrina, a disease of equine animals in the Isthmus, is treated by S. T. Darling. It occurs in oedematous and paretic types, the former being called murrina by the natives and being always rather chronic; while the latter is termed derrengadera and may be either acute or chronic. While apparently distinct clinically, both are due to one trypanosome, T. hippicum, probably introduced by a fly. All of the various mammalian trypanosomes are excluded by differences in incidence, morphology, etc., easily, except T. equinum, the cause of mal de caderas, also a South American equine epizootic, but for various reasons, Darling considers this distinct. While work horses and mules were the only subjects of natural infection, artificial inoculation succeeded in the saddle horse, pig, dog, raccoon, cat, two kinds of monkeys, rabbit, guinea pig, four species of rat and two of mouse, opossum, agouti and coati. The calf was refractory.

